# AI-Based Facial Emotion Analysis for Early and Differential Diagnosis of Dementia

**DOI:** 10.3390/bioengineering12101082

**Published:** 2025-10-04

**Authors:** Letizia Bergamasco, Anita Coletta, Gabriella Olmo, Aurora Cermelli, Elisa Rubino, Innocenzo Rainero

**Affiliations:** 1 Department of Control and Computer Engineering, Politecnico di Torino, 10129 Turin, Italy; anita.coletta@polito.it (A.C.); gabriella.olmo@polito.it (G.O.); 2 LINKS Foundation, 10138 Turin, Italy; 3 Center for Alzheimer’s Disease and Related Dementias, Department of Neuroscience and Mental Health, A.O.U. Città Della Salute e Della Scienza di Torino, 10126 Turin, Italy; aurora.cermelli@unito.it (A.C.); elisa.rubino@unito.it (E.R.); innocenzo.rainero@unito.it (I.R.); 4 Department of Neuroscience “Rita Levi Montalcini”, University of Torino, 10126 Turin, Italy

**Keywords:** artificial intelligence, machine learning, facial expression recognition, affective computing, mild cognitive impairment, dementia, Alzheimer’s disease

## Abstract

Early and differential diagnosis of dementia is essential for timely and targeted care. This study investigated the feasibility of using an artificial intelligence (AI)-based system to discriminate between different stages and etiologies of dementia by analyzing facial emotions. We collected video recordings of 64 participants exposed to standardized audio-visual stimuli. Facial emotion features in terms of valence and arousal were extracted and used to train machine learning models on multiple classification tasks, including distinguishing individuals with mild cognitive impairment (MCI) and overt dementia from healthy controls (HCs) and differentiating Alzheimer’s disease (AD) from other types of cognitive impairment. Nested cross-validation was adopted to evaluate the performance of different tested models (K-Nearest Neighbors, Logistic Regression, and Support Vector Machine models) and optimize their hyperparameters. The system achieved a cross-validation accuracy of 76.0% for MCI vs. HCs, 73.6% for dementia vs. HCs, and 64.1% in the three-class classification (MCI vs. dementia vs. HCs). Among cognitively impaired individuals, a 75.4% accuracy was reached in distinguishing AD from other etiologies. These results demonstrated the potential of AI-driven facial emotion analysis as a non-invasive tool for early detection of cognitive impairment and for supporting differential diagnosis of AD in clinical settings.

## 1. Introduction

Dementia is a term used to describe a syndrome characterized by a progressive deterioration of cognitive functions and behavioral disturbances. The World Health Organization (WHO) acknowledges it as one of the main causes of disability and loss of autonomy in the elderly population at a global level [1]. Alzheimer’s disease (AD) is the most common form of neurodegenerative dementia, typically presenting with short-term memory impairment at the onset [2]. Less frequent types of dementia include vascular dementia (VD) [3], frontotemporal dementia (FTD) [4], dementia with Lewy bodies (DLB) [5], and mixed forms.

Early and differential diagnosis is essential for timely access to care and support for dementia, as well as for enrolling individuals in clinical trials. At present, therapeutic approaches primarily aim to provide symptomatic relief [6]. However, the Food and Drug Administration has recently approved disease-modifying therapies for AD [7,8], showing clinical efficacy only when administered during the earliest stages of the disease.

From a clinical perspective, Alzheimer’s disease is a progressive continuum that begins with an asymptomatic phase, progresses to a stage of mild cognitive impairment (MCI), and ultimately culminates in the onset of overt dementia [9]. In the MCI stage, the first symptoms occur, but most patients can live independently and their daily activities are not heavily affected. Hence, this stage is often considered an important time window for detecting and diagnosing cognitive decline at an early stage.

The diagnosis of MCI and dementia commonly relies on a combination of medical history, neuropsychological assessments, neuroimaging, and laboratory tests, which are often costly, invasive, and require specialized clinical expertise. Therefore, there is a pressing need for the development of accessible, cost-effective approaches for the early detection of cognitive impairment (CI). In this context, facial expressions may carry important diagnostic information; indeed, altered facial expressivity is commonly observed in individuals with CI, and such alterations tend to differ depending on the type and stage of dementia [10,11]. This makes the assessment of facial emotional expression potentially useful for distinguishing between different forms of dementia.

Computer vision approaches, especially those based on deep learning (DL), have shown considerable potential in analyzing facial expressions to support early and accurate diagnosis of CI. Some studies have adopted end-to-end DL models trained on facial images. Sun et al. [12] achieved an accuracy of 90.63% in distinguishing MCI from healthy controls (HCs) using a Multi-branch Classifier–Video Vision Transformer. The study employed a subset of the I-CONECT dataset (83 MCI and 64 HCs) [13], including semi-structured interviews. Umeda-Kameyama et al. [14] achieved 92.56% accuracy in CI detection with an Xception DL model on a dataset comprising 484 face images, including 121 patients with AD and 117 HCs.

Other studies extracted facial-related features to perform CI detection. Zheng et al. [15] achieved a dementia detection accuracy of 79% with Histogram of Oriented Gradients features; an accuracy of 71% with Action Units (AUs); and an accuracy of 66% with face mesh features. The authors employed a subset of the PROMPT dataset [16], encompassing 447 videos from 117 subjects, including HC and dementia patients. Facial features representing emotions are of particular interest, not only for exhibiting good performance, but also for facilitating model interpretability for clinicians, since emotional regulation is directly affected by dementia. Fei et al. [17] extracted categorical emotion features with a DL-based model and used them to train a Support Vector Machine (SVM), reaching a 73.3% accuracy in distinguishing between subjects with CI and HCs. The authors enrolled 61 elderly people (36 CI and 25 HCs) for the study dataset and developed an interface to record facial videos while displaying emotional video stimuli. In a previous study from our research group [18], we proposed a novel approach to CI detection; while our aim was similar to that of [17], we integrated a dimensional model of affect for a more comprehensive representation of emotions, and we adopted standardized emotion-eliciting stimuli for data collection. We achieved a 76.7% accuracy in CI detection from facial videos recorded from 60 subjects (32 CI and 28 HCs).

The above-mentioned studies focused specifically on the detection of MCI or dementia, or targeted CI detection by grouping subjects with MCI and dementia. However, very few works in the literature tackled the differentiation of different stages of the disease combined (e.g., MCI and overt dementia) or different underlying etiologies (e.g., AD and other forms of neurodegenerative conditions). A recent work by Okunishi et al. [19] proposed a method to detect MCI and dementia based on AUs, eight emotion categories, valence–arousal, and face embeddings. By extracting and combining all these features from video recordings, they achieved an 86.2% accuracy for dementia detection and an 83.4% accuracy for MCI detection on a selected subset of the PROMPT dataset [16]. Chu et al. [20] recruited 95 participants (41 MCI; 54 mild-to-moderate dementia) and recorded them while administering the Short Portable Mental Status Questionnaire. They performed a binary classification of MCI and dementia with DL models trained on visual and speech features, reaching a 76.0% accuracy (rising to 88% when excluding depression and anxiety). On the other hand, Jiang et al. [21] conducted a comprehensive study involving 493 individuals who were video-recorded while undergoing a passive memory test. The cohort included HCs, individuals with MCI due to AD, individuals with MCI due to other etiologies, individuals with dementia due to AD, individuals with dementia due to other etiologies, and individuals with subjective CI. Employing DL-based facial emotion analysis, the authors successfully differentiated individuals with CI from HCs; however, they were unable to differentiate among the underlying etiologies. Moreover, the study revealed that CI subjects display fewer positive emotions, more negative emotions, and greater facial expressiveness compared to HCs.

Based on our previous work [18], the main objectives of this work are as follows:

- To explore the automatic detection of both MCI and overt dementia employing elicited facial emotion features extracted from video recordings;
- To assess the capability of the proposed system to discriminate AD from other forms of cognitive impairment. To the best of our knowledge, this is the first study to propose an automated method to differentiate between diverse etiologies of dementia based on facial emotion analysis.

For these purposes, we set up an emotion elicitation protocol and collected video recordings from subjects whose clinical diagnosis was supported by relevant biomarkers, including AD-specific biomarkers in the cerebrospinal fluid. The obtained results demonstrate that the proposed system achieves good performance not only in detecting CI, but also in identifying MCI patients and discriminating AD, thus showing promising support for the early and differential diagnosis of dementia.

The remainder of the paper is structured as follows. Section 2 describes the collected dataset, the architecture of the employed system for CI detection, and the experiments conducted; Section 3 presents the experimental results; and, finally, Section 4 discusses the results and outlines future research directions.

## 2. Materials and Methods

### 2.1. Collected Data

The dataset encompassed video recordings from subjects exposed to an emotion-eliciting video. The full protocol used for data collection was previously introduced in [18]. In detail, the emotion-eliciting video was created using images and sounds from two databases widely used in affective stimulation research, i.e., IAPS (International Affective Picture System [22]) and IADS-2 (International Affective Digitized Sounds-2 [23]). The identification numbers of the selected picture–-sound pairs from IAPS and IADS-2, respectively, were as follows: [8501, 367], [8185, 817], [8030, 352], [8190, 815], [8370, 363], [8492, 360], [5760, 811], [5000, 812], [2035, 810], [1441, 809], [2360, 151], [2530, 230], [9075, 260], [9410, 286], [9635.1, 292], [3530, 276], [3005.1, 296], [2750, 250], [9342, 382], [9280, 701], [9832, 728], [9220, 723], [7031, 708], [8232, 364], [1908, 170], [9422, 410], and [2780, 722]. This ensured the standardization of the emotional stimuli adopted in the protocol. Specifically, 28 pairs consisting of an image and a sound with similar valence and arousal were displayed in a fixed and randomly selected sequence. To ensure the safety and comfort of participants, particularly given the clinical vulnerability of the recruited patients, the selection of audio-visual stimuli was made in consultation with clinicians. High-impact or emotionally intense stimuli from the IAPS and IADS-2 databases were deliberately avoided. As a result, the elicited valence and arousal values tended not to reach extreme levels, but the adopted protocol ensured ethical suitability and tolerability within this fragile population.

Following the protocol, participants were seated in front of a laptop that simultaneously showed the emotion-eliciting video and recorded the facial expressions using an external USB webcam (Logitech C920, 1080p resolution, 30 fps). The adopted frame rate was deemed adequate for our purposes, since emotions are also related to rapid microexpressions, with duration of less than 200 ms [24]. A nearby external Bluetooth speaker was used to ensure high-quality sound output. The experiment was set up using PsychoPy v2022.2.4 software [25], which allowed for the synchronized presentation of emotional stimuli and simultaneous webcam recording.

The emotion-eliciting video had a duration of approximately 8 min. It began with a 10 s webcam calibration stage, followed by a welcome title (duration of 5.5 s). Then, the sequence of 28 audio-visual stimuli followed: each trial started with a 10 s countdown, followed by a 1 s display of a central cross. Subsequently, an image was presented for 6 s while simultaneously playing the corresponding sound. Once the audio-visual stimuli were completed, the recording was stopped, and a concluding title was displayed for 1 s.

**Figure 1:**
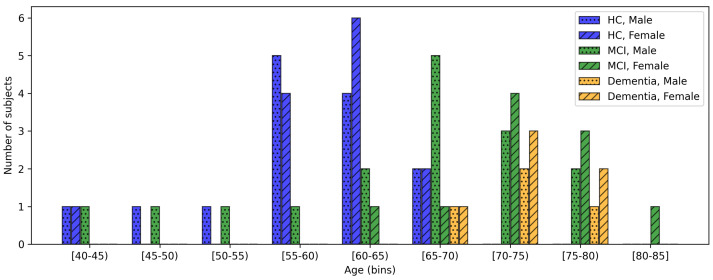
Age distribution of participants across clinical groups (HCs, MCI, and dementia) and sex.

Starting from the data presented in [18], we collected additional video recordings to expand the database. In total, data from 64 participants were obtained, including 28 HCs, 26 subjects diagnosed with MCI (13: due to AD; 13: other types), and 10 diagnosed with overt dementia (4: AD; 6: other types). Diagnoses of AD were performed according to the NIA-AA (National Institute on Aging and the Alzheimer’s Association) AT(N) criteria, incorporating biomarkers for amyloid (A), tau (T), and neurodegeneration (N) [26,27,28]. Non-AD forms of CI included different etiologies such as FTD, DLB, VD, and mixed or not specified forms; individuals with subjective CI were also included. Diagnoses of FTD, DLB, VD and other neurocognitive disorders were made based on established diagnostic criteria specific to each condition [29,30,31,32]. The experiments were conducted in a designated room at Molinette Hospital–A.O.U. Città della Salute e della Scienza di Torino. Table 1 provides a summary of the demographic characteristics and key clinical information. Moreover, Figure 1 shows the age distribution of participants across clinical groups (HCs, MCI, and dementia) and sex. To protect participants’ privacy, all data were pseudonymized using unique participant codes, and all metadata were stored separately from sensitive information. Original video recordings were stored on secured institutional servers, with access restricted to authorized personnel only.

CI participants were selected from individuals seeking a diagnosis of cognitive disorders at the Center for Alzheimer’s Disease and Related Dementias of the Department of Neuroscience and Mental Health, A.O.U. Città della Salute e della Scienza University Hospital (Turin, Italy). The diagnoses and differential diagnoses of CI were conducted following a comprehensive neurological and neurocognitive evaluation, including neuropsychological testing, brain imaging (MRI and 18F-fluorodeoxyglucose PET), and lumbar puncture for cerebrospinal fluid biomarker analysis (Aβ42, Aβ42/Aβ40, total tau, and phosphorylated tau 181). Exclusion criteria encompassed minors (<18 years), individuals lacking legal capacity, and any additional condition which, in the judgment of the investigators, would interfere with the patient’s ability to comply with the study protocol or would render the patient ineligible (e.g., patients with significant motor limitations affecting facial expressions).

The classification between subjects with MCI and overt dementia was based on cognitive evaluations conducted by a trained neuropsychologist (A.Ce.), using the Mini-Mental State Examination (MMSE), the Montreal Cognitive Assessment (MoCA), and the assessments of functional independence through the Activities of Daily Living (ADLs) and Instrumental Activities of Daily Living (IADLs) scales. CI participants were classified as MCI-affected if they met the criteria of MMSE ≥20, ADL =6/6, and IADL ≥6/8. Those with MMSE <20 or ADL <6/6 or IADL <6/8 were categorized as overt dementia patients.

HC subjects were volunteers aged between 40 and 80 years. Exclusion criteria included the presence of neurological or psychiatric disorders, as well as any other condition that could hinder participation in the experiment (e.g., blindness). All HC subjects underwent neuropsychological assessment to confirm eligibility, which required an MMSE ≥26/30, ADL =6/6, and IADL =8/8.

### 2.2. System Architecture and Data Processing

**Figure 2:**
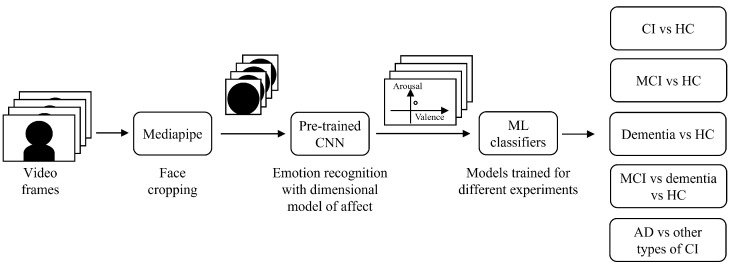
Schematic overview of the proposed system architecture.

The experiments relied on an architecture based on the system presented in [18], adapted to perform different CI detection tasks. The systems consists of two subsequent parts: (i) obtaining the evolution of the emotions from the collected videos, in terms of valence and arousal, and (ii) using the emotion data to train a machine learning (ML) model for the selected CI detection task. A schematic overview of the developed system is provided in Figure 2.

For each video, all frames were extracted (∼14 k); then, the subject’s face was cropped using the MediaPipe [33] Holistic solution, with a 224×224 pixel size. Each frame was processed by two pre-trained Convolutional Neural Networks (CNNs) to perform facial emotion recognition and estimate valence and arousal values, respectively. Emotions were therefore represented with a dimensional model, namely the circumplex model [34], using a circular space defined by two affect dimensions: valence, indicating whether an emotion is positive or negative, and arousal, indicating its intensity. With respect to categorical models of emotions (e.g., Ekman’s Basic Emotions model [35] with six basic emotions), dimensional models allow capturing all possible emotion nuances.

The CNN models, introduced and detailed in [18], were trained on AffectNet [36], a large and diverse in-the-wild facial expression dataset. While it is built from the general population, AffectNet remains a widely used and practical starting point given the lack of large emotion datasets including CI individuals. Both CNNs were based on a Squeeze-and-Excitation Network (SENet) architecture pre-trained on VGGFace2 [37] and fine-tuned on AffectNet [38], following the transfer learning approach introduced in [18]. The original classification head of the pre-trained SENet model [39] was replaced with a global average pooling layer and two fully connected layers (2048 and 1024 units), followed by three output branches: one for categorical emotion classification (8 neurons) and two for valence and arousal prediction (1 neuron each). Although the target attributes were valence and arousal, respectively, the categorical branch was included during training to support a multi-task learning (MTL) setup [40], which provided improved performance with respect to single-task learning (i.e., learning emotional attributes separately) [18]. Each CNN model was trained using a weighted loss function combining Mean Squared Error for valence and arousal and Cross-Entropy loss for categorical expressions. Loss weights were tuned to optimize the target attribute (valence or arousal, respectively), resulting in two final CNNs—one optimized for valence prediction and one optimized for arousal prediction—with both leveraging shared learning. Training was performed on over 280 k face images (with 5% reserved for validation), using batch size 32, adaptive learning rate, early stopping, and the Adam optimizer. Data augmentation included random horizontal flipping and rotation (±20 degrees). The training of the CNNs was performed on a locally hosted server featuring an NVIDIA GeForce RTX 3080 GPU, an Intel i9-10900X processor, and 64 GB of RAM. The experiments were conducted using Python version 3.10.16, TensorFlow and Keras [41] version 2.9.0, with CUDA version 12.2. The two CNNs were tested on an independent test set of ∼4 k images, showing a performance on valence and arousal prediction comparable to the AffectNet benchmark [36]. Hence, these CNN models were deemed adequate to be used within our system for CI detection. The exploration of more complex DL models for facial emotion recognition was beyond the scope of this paper and is left to future work.

The resulting valence and arousal series were concatenated into a single feature vector, representing the changes in the emotional states of the participants throughout the experiment. These feature vectors were used to train different classification algorithms (detailed in Section 2.4) according to the experiments, as discussed in the following section.

### 2.3. Experiments

With the same system pipeline outlined in Section 2.2, five different experiments were performed.

1. *CI vs. HCs*: In this experiment, all CI subjects were grouped together and compared to the HC group through a binary classification task. This allowed for the validation of the generalization capability of the proposed algorithm when tested on the expanded dataset, compared to that in [18]. The dataset included 64 subjects in total: 36 CI (26 MCI + 10 overt dementia) and 28 HCs.
2. *MCI vs. HCs*: For this experiment, only subjects with a clinical diagnosis of MCI were selected among the CI group. The objective was to investigate whether any differences from the HC group would be detected during the earlier stages of the disease. The dataset included 54 subjects: 26 MCI and 28 HCs. A binary classification task was applied to distinguish between these two classes.
3. *Dementia vs. HCs*: In contrast to the previous experiment, this analysis included only patients with overt dementia, with the aim of identifying the differences from the HC group appearing during the later stages of the disease. The dataset included 38 subjects: 10 overt dementia and 28 HCs. A binary classification task was carried out to distinguish between these two classes.
4. *MCI vs. dementia vs. HCs*: In this experiment, the three different classes of subjects were compared, according to the level of severity of the disease. The dataset included 64 subjects in total: 26 MCI, 10 with overt dementia, and 28 HCs. The analysis moved from a binary to a multiclass classification task among the three classes. It should be noted that the dataset was imbalanced across classes, with the overt dementia group including fewer subjects compared to the other two.
5. *AD vs. other types of CI*: The aim of this last experiment was to investigate any differences in facial emotion responses among individuals with different types of CI. Specifically, patients diagnosed with AD were grouped together and compared to the broader group of individuals with other forms of CI. This approach was motivated by the fact that AD is the most common cause of dementia, and a differential diagnosis distinguishing AD from other etiologies is of critical clinical importance. The dataset included 36 subjects: 26 MCI (13: due to AD; 13: other types) and 10 subjects with overt dementia (4: AD, 6: other types). Two classes were considered: AD (17 subjects) and other types of CI (19 subjects). A binary classification task was performed to distinguish between these two classes.

### 2.4. Model Selection and Evaluation

Machine Learning classifiers were implemented using the scikit-learn Python library [42]. For the binary classification tasks (experiments 1, 2, 3, 5), also considering the limited size of our dataset, K-Nearest Neighbors (KNN), Logistic Regression (LR), and Support Vector Machine (SVM) models were selected. The KNN model was optimized through a grid search approach over the number of neighbors (3, 5, 7) and the choice of distance metric (Euclidean, Manhattan, and Chebyshev). For LR, we applied an L2 regularization term with the “liblinear” solver, a tolerance for stopping criteria of $10^{-4}$ and tuned the inverse of the regularization strength parameter *C* across a range of powers of 10, from $10^{-4}$ to $10^{4}$. The SVM was configured with a linear kernel, as well as a tolerance of $10^{-3}$, and was similarly tuned on the regularization parameter *C*, with powers of 10 ranging from $10^{-4}$ to $10^{4}$.

For the multiclass classification task (experiment 4), the ML models were implemented similarly, with a few adjustments to accommodate the multiclass setting. As the scikit-learn KNN estimator supports multiclass problems, no modifications were required. For the LR estimator, the “liblinear” solver was changed to “lbfgs” to handle multinomial loss. In the SVM estimator, multiclass classification is managed using a one-vs-one strategy, implemented following the same procedure used for the binary classification tasks.

A nested cross-validation (NCV) technique was adopted to provide an unbiased estimate of the generalization error of the models. Indeed, with limited size datasets, the standard cross-validation (CV) used for both hyperparameter tuning and performance estimation may produce over-optimistic results. Instead, NCV uses an inner CV loop to search for the best set of model parameters and an outer CV loop to evaluate the final model performance independently. This separation leads to a less biased and more realistic estimate of the generalization error of the model. In all experiments, a 5-fold CV was used in the outer loop, and a 3-fold CV was used in the inner loop. This configuration was chosen in line with standard practice to achieve a good balance between bias and variance: it provides reliable performance estimates while ensuring that each fold includes a sufficient amount of data for training. To maintain class distribution consistency across folds, both the outer and inner CV loops used a stratified k-fold cross-validation, as implemented in scikit-learn, ensuring that each fold contained approximately the same class proportions as the original dataset. Stratification was applied at the participant level, i.e., all recordings from the same individual were allocated exclusively to either the training or the validation set within each fold, avoiding data leakage between folds. This procedure was applied to all the ML models under consideration. The model achieving the highest average accuracy across the NCV outer folds was selected as the best-performing one; on the selected model, the optimal set of hyperparameters was determined as the most frequently chosen combination across the outer folds.

## 3. Results

The results obtained in the different classification experiments involving CI and HC subjects (experiments 1, 2, 3, 4) are shown in Table 2. In summary, when classifying all CI subjects vs. HCs, the best-performing model was the KNN model, with a 73.6% accuracy and an F1 score of 72.2%. When considering different stages of CI separately, another KNN model reached the highest accuracy of 76.0% and an F1 score of 74.5% when classifying MCI versus HC subjects. On the other hand, an SVM reached the best accuracy of 73.6% in distinguishing dementia from HC subjects. However, in this experiment, the F1 scores for all the tested models were lower, reflecting a tendency to misclassify dementia subjects, likely due to class imbalance and limited sample size. Lastly, for the multiclass problem (MCI, dementia, and HCs), a cross-validation accuracy of 64.1% was reached by a KNN model. F1 scores in this setting remained relatively low.

**Figure 3:**
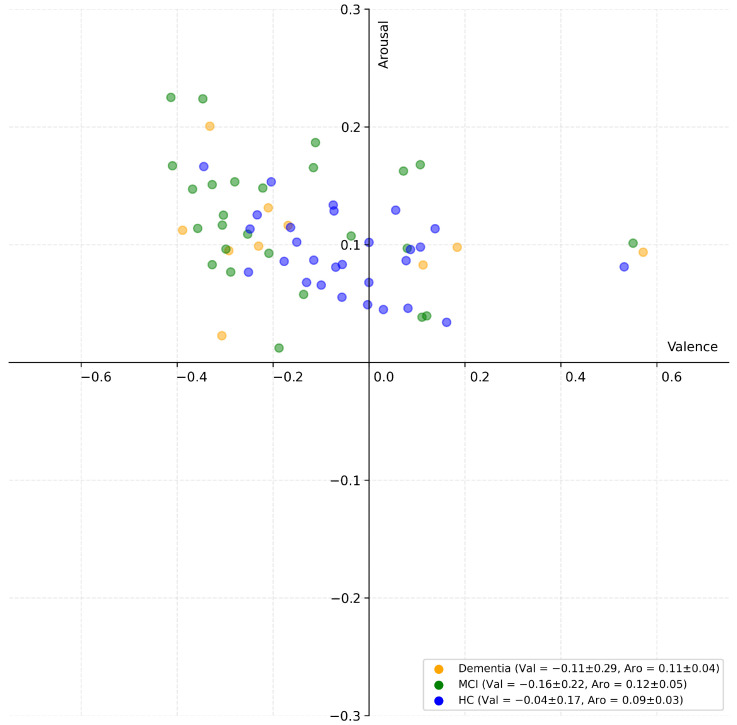
Scatter plot of valence and arousal values averaged across all frames of each video. The legend also reports the group-level summary statistics (mean ± standard deviation of valence and arousal) for the HC, MCI, and dementia groups.

To complement the quantitative analyses, a scatter plot of valence and arousal values averaged across each video was generated to provide an intuitive visualization of the emotional information contained in the feature vectors (Figure 3). The obtained valence–arousal distribution was consistent with the experimental design: given the deliberate avoidance of highly intense stimuli to ensure the safety and comfort of participants, the elicited responses naturally clustered around moderate values, without reaching extreme levels. This constrained affective space contributing to the observed overlap among groups, although some group-level differences could still be detected. On average, both the MCI and dementia groups displayed slightly lower valence values compared to HCs, whereas arousal values remained comparable across the three groups. Overall, this visual overlap underscored the intrinsic difficulty of the classification task and highlighted the necessity of ML approaches to capture more subtle and multidimensional patterns in the data.

Table 3 reports the results of the classification between AD and other types of CI (experiment 5). As observable, the KNN model achieved the best accuracy of 75.4% and an F1 score of 74.9%.

## 4. Discussion

The results obtained in this study suggest that the employed models are capable of distinguishing CI and HC subjects using solely facial emotion data collected with our protocol (Table 2). With respect to [18] (reporting a 76.7% accuracy for the classification of CI vs. HC subjects), the present study, based on an enlarged dataset, achieved a comparable accuracy of 73.6%, suggesting that the method maintained good performance despite increased heterogeneity within the CI population. Nevertheless, further data collection is needed to more comprehensively assess the generalizability of these findings.

The results obtained for CI detection support the potential of emotional features extracted from recorded video data as an effective tool to aid CI screening. This further highlights the value of exploring the emotional dimension in the context of early diagnosis, an area currently underexplored in standard neuropsychological assessments such as the MMSE and MoCA, yet shown to be informative in previous studies [17,18,19,21]. Recently, a review by Alsuhaibani et al. [43] explored emerging deep learning approaches for non-invasive cognitive impairment detection, covering a range of modalities including speech, facial expressions, motor behavior, and eye movements. While speech-based methods achieved the highest performance overall, especially those combining acoustic and linguistic features, facial analysis techniques were identified as a promising yet underexplored direction, requiring further investigation to establish their reliability. These findings reinforce the relevance of our focus on emotional facial features as valuable indicators of cognitive status and motivate the approach proposed in our work.

When analyzing the classification performance across different stages of cognitive decline (specifically, MCI vs. HCs and dementia vs. HCs), the accuracy was comparable to, or even higher than, that observed in the previous experiment, with values of 76.0% and 73.6%, respectively. The observed difference between the MCI vs. HC (76.0%) and dementia vs. HC (73.6%) accuracies should be interpreted cautiously, as a more reliable comparison would require similar sample sizes for both groups. Nevertheless, these preliminary results demonstrate satisfactory accuracy when focusing exclusively on MCI patients, without those with overt dementia. Notably, the F1 scores were consistently lower for the dementia vs. HC classification, suggesting a greater tendency toward misclassification in the dementia group. This may be due to class imbalance and the greater heterogeneity of facial expressions in more advanced stages of cognitive decline. However, the enrollment of overt dementia patients is difficult, especially in advanced stages. Overall, these findings suggest that the proposed method holds promise for supporting the early diagnosis of cognitive impairment, as it performed effectively in subjects at an earlier stage of CI.

Regarding the multiclass classification task (MCI vs. dementia vs. HCs), the accuracy decreased compared to the binary classification tasks (MCI vs. HCs and dementia vs. HCs), yet it remained relatively high (64.1%) considering the increased complexity of the problem. This outcome is not surprising, as distinguishing CI individuals from HCs is generally easier than the more fine-grained differentiation between different stages within the dementia continuum. In addition, the F1 scores obtained in the multiclass setting were lower overall, indicating greater classification uncertainty and class imbalance effects when attempting to separate three groups simultaneously. Interestingly, this limitation was also observed in studies applying ML to neuroimaging data for the diagnosis and prognosis of CI and dementia. In fact, Pellegrini et al. [44] reported that although ML methods achieve an acceptable accuracy in distinguishing overt AD from HCs, their performance drops when tackling the differentiation of MCI from AD, MCI from HCs, or the prediction of MCI conversion to AD.

One of the most notable findings of this study regards the discrimination of AD vs. other types of CI, reaching a cross-validation accuracy of 75.4% (Table 3). This result is particularly promising, suggesting that a differential diagnosis of AD might be feasible through a non-invasive approach, for example, by exploiting facial emotion analysis. While preliminary, this approach may serve as a complementary aid to traditional diagnostic methods, offering a non-invasive and accessible tool to support early clinical assessment.

A comprehensive performance comparison with related studies is currently challenging, due to the differences in the video datasets used, the corresponding data collection protocols, and the varying definitions of the ML classification tasks. A distinguishing feature of the present work, compared to others such as [17,21], lies in the use of an emotion-eliciting protocol based on standardized and extensively validated stimuli [22,23], enhancing its ease of adoption and generalizability. Moreover, our study adopted a dimensional model of emotions, providing a more comprehensive characterization of affective states compared to the categorical approach used by Fei et al. [17], who also focused on CI detection based solely on facial emotions. Most importantly, one of the key strengths of the study is the availability of well-characterized ground truth classifications for CI subjects. Indeed, unlike other studies where the subjects’ type of CI was often assumed and not properly confirmed [14,20], in our study, the diagnosis process was based on a comprehensive evaluation, as explained in Section 2.1, and AD diagnoses were supported by biomarkers from cerebrospinal fluid.

While the experimental results were promising, this study presents with some limitations. First, the collected dataset had a limited sample size (64 participants), exclusively including individuals of Caucasian ethnicity and recruited from a single clinical center within a specific setting. To further validate the generalization capability of the presented results, future work will focus on expanding the dataset, possibly including diverse ethnicities, and ensuring a multicenter perspective. This will also enable deeper investigation by incorporating subject stratification based on demographic and clinical characteristics, as well as validation on independent cohorts. Second, the dataset was imbalanced across classes, with the dementia group including less subjects than the MCI and the HCs. This imbalance resulted from the recruitment process, in which patients were enrolled based on eligibility criteria during outpatient visits to the Center for Alzheimer’s Disease and Related Dementias, without prior knowledge of their clinical diagnosis. Additional experiments with larger datasets and the adoption of more advanced techniques to address class imbalance will be performed in future studies. Future developments will also investigate suitable data augmentation strategies to address the limitations related to sample size and variability.

In addition, the overall system performance was largely influenced by the method used for facial emotion feature extraction, which still offers potential for improvement and could allow for a more accurate encoding of emotional information. Therefore, future research directions include the exploration of more complex DL architectures that demonstrated good performance in facial emotion recognition tasks and related domains. These include approaches incorporating attention mechanisms [45,46], Visual Transformers [47,48], and Bidirectional Long Short-Term Memory (BiLSTM) networks [49]. Hybrid architectures combining BiLSTMs, attention mechanisms, and Kolmogorov–Arnold Networks have also been explored in other fields [50], offering inspiration for analyzing complex multimodal emotional patterns in future developments. Additionally, we plan to investigate the integration of a wider range of facial features beyond valence and arousal, as the combination of multiple feature types was shown to be effective in previous studies [19]. We also deem it important to further explore in future work the temporal dynamics and the individual role of each affective component; such work may reveal additional informative patterns of emotional responses relevant for the detection of cognitive impairment and enhancing clinical applicability.

## Figures and Tables

**Table 1:** Participants’ demographics and key clinical information.

	MCI	Overt Dementia	Healthy Controls
**Number of subjects**	26	10	28
**Age** (mean ± standard deviation)	68.2 ± 9.3	72.9 ± 3.8	58.8 ± 6.9
**Sex** (number of females, %)	10 (38.5%)	6 (60.0%)	14 (50.0%)
**Ethnicity**	Caucasian	Caucasian	Caucasian
**Years of education** (mean ± standard deviation)	13.7 ± 4.6	10.4 ± 5.4	15.6 ± 4.8
**MMSE score** (mean ± standard deviation)	25.8 ± 3.6	18.8 ± 5.5	29.2 ± 1.2
**MoCA score** (mean ± standard deviation)	20.0 ± 4.4	14.0 ± 3.6	25.4 ± 2.2
**Differential CI diagnosis**	13: due to AD; 13: other types	4: AD; 6: other types	No cognitive impairment

**Table 2:** Cross-validation performance for different classification experiments involving CI and HC subjects (mean ± standard deviation).

Experiment	Model	Parameters	Accuracy	F1 Score
CI vs. HCs	KNN	3 neighbors, Manhattan distance	**0.736 ± 0.102**	**0.722 ± 0.111**
	LR	L2 penalty, tolerance = 0.0001, C = 0.001	0.623 ± 0.139	0.620 ± 0.141
	SVM	linear kernel, tolerance = 0.001, C = 0.01	0.624 ± 0.092	0.612 ± 0.092
MCI vs. HCs	KNN	3 neighbors, Manhattan distance	**0.760 ± 0.041**	**0.745 ± 0.048**
	LR	L2 penalty, tolerance = 0.0001, C = 0.001	0.684 ± 0.114	0.674 ± 0.110
	SVM	linear kernel, tolerance = 0.001, C = 0.001	0.667 ± 0.069	0.664 ± 0.068
Dementia vs. HCs	KNN	3 neighbors, Euclidean distance	0.732 ± 0.097	0.487 ± 0.156
	LR	L2 penalty, tolerance = 0.0001, C = 0.1	0.654 ± 0.145	0.492 ± 0.174
	SVM	linear kernel, tolerance = 0.001, C = 0.0001	**0.736 ± 0.018**	0.424 ± 0.006
MCI vs. dementia vs. HCs	KNN	5 neighbors, Manhattan distance	**0.641 ± 0.103**	0.463 ± 0.076
	LR	L2 penalty, tolerance = 0.0001, C = 0.01	0.591 ± 0.104	0.427 ± 0.109
	SVM	linear kernel, tolerance = 0.001, C = 0.1	0.578 ± 0.077	0.413 ± 0.051

**Table 3:** Cross-validation performance for the classification of AD versus other types of CI (mean ± standard deviation).

Experiment	Model	Parameters	Accuracy	F1 Score
AD vs. other types of CI	KNN	5 neighbors, Chebyshev distance	**0.754 ± 0.128**	**0.749 ± 0.130**
	LR	L2 penalty, tolerance = 0.0001, C = 0.0001	0.586 ± 0.171	0.571 ± 0.174
	SVM	linear kernel, tolerance = 0.001, C = 0.01	0.643 ± 0.090	0.602 ± 0.088

## Data Availability

The dataset presented in this article is not readily available due to privacy restrictions.

## References

[1] World Health Organization (2021). Global Status Report on the Public Health Response to Dementia.

[2] Scheltens P., Blennow K., Breteler M.M., De Strooper B., Frisoni G.B., Salloway S., Van der Flier W.M. (2016). Alzheimer’s disease. Lancet.

[3] T O’Brien J., Thomas A. (2015). Vascular dementia. Lancet.

[4] Bang J., Spina S., Miller B.L. (2015). Frontotemporal dementia. Lancet.

[5] Walker Z., Possin K.L., Boeve B.F., Aarsland D. (2015). Lewy body dementias. Lancet.

[6] Koyama A., Okereke O.I., Yang T., Blacker D., Selkoe D.J., Grodstein F. (2012). Plasma amyloid-*β* as a predictor of dementia and cognitive decline: A systematic review and meta-analysis. Arch. Neurol..

[7] Alexander G.C., Emerson S., Kesselheim A.S. (2021). Evaluation of aducanumab for Alzheimer disease: Scientific evidence and regulatory review involving efficacy, safety, and futility. JAMA.

[8] van Dyck C.H., Swanson C.J., Aisen P., Bateman R.J., Chen C., Gee M., Kanekiyo M., Li D., Reyderman L., Cohen S. (2023). Lecanemab in Early Alzheimer’s Disease. N. Engl. J. Med..

[9] Albert M.S., DeKosky S.T., Dickson D., Dubois B., Feldman H.H., Fox N.C., Gamst A., Holtzman D.M., Jagust W.J., Petersen R.C. (2011). The diagnosis of mild cognitive impairment due to Alzheimer’s disease: Recommendations from the National Institute on Aging-Alzheimer’s Association workgroups on diagnostic guidelines for Alzheimer’s disease. Alzheimer’s Dement..

[10] Chen K.H., Lwi S.J., Hua A.Y., Haase C.M., Miller B.L., Levenson R.W. (2017). Increased subjective experience of non-target emotions in patients with frontotemporal dementia and Alzheimer’s disease. Curr. Opin. Behav. Sci..

[11] Pressman P.S., Chen K.H., Casey J., Sillau S., Chial H.J., Filley C.M., Miller B.L., Levenson R.W. (2023). Incongruences between facial expression and self-reported emotional reactivity in frontotemporal dementia and related disorders. J. Neuropsychiatry Clin. Neurosci..

[12] Sun J., Dodge H.H., Mahoor M.H. (2024). MC-ViViT: Multi-branch Classifier-ViViT to detect Mild Cognitive Impairment in older adults using facial videos. Expert Syst. Appl..

[13] Dodge H.H., Yu K., Wu C.Y., Pruitt P.J., Asgari M., Kaye J.A., Hampstead B.M., Struble L., Potempa K., Lichtenberg P. (2023). Internet-Based Conversational Engagement Randomized Controlled Clinical Trial (I-CONECT) Among Socially Isolated Adults 75+ Years Old with Normal Cognition or Mild Cognitive Impairment: Topline Results. Gerontologist.

[14] Umeda-Kameyama Y., Kameyama M., Tanaka T., Son B.K., Kojima T., Fukasawa M., Iizuka T., Ogawa S., Iijima K., Akishita M. (2021). Screening of Alzheimer’s disease by facial complexion using artificial intelligence. Aging.

[15] Zheng C., Bouazizi M., Ohtsuki T., Kitazawa M., Horigome T., Kishimoto T. (2023). Detecting Dementia from Face-Related Features with Automated Computational Methods. Bioengineering.

[16] Kishimoto T., Takamiya A., Liang K., Funaki K., Fujita T., Kitazawa M., Yoshimura M., Tazawa Y., Horigome T., Eguchi Y. (2020). The project for objective measures using computational psychiatry technology (PROMPT): Rationale, design, and methodology. Contemp. Clin. Trials Commun..

[17] Fei Z., Yang E., Yu L., Li X., Zhou H., Zhou W. (2022). A Novel deep neural network-based emotion analysis system for automatic detection of mild cognitive impairment in the elderly. Neurocomputing.

[18] Bergamasco L., Lorenzo F., Coletta A., Olmo G., Cermelli A., Rubino E., Rainero I. (2025). Automatic Detection of Cognitive Impairment Through Facial Emotion Analysis. Appl. Sci..

[19] Okunishi T., Zheng C., Bouazizi M., Ohtsuki T., Kitazawa M., Horigome T., Kishimoto T. (2025). Dementia and MCI Detection Based on Comprehensive Facial Expression Analysis From Videos During Conversation. IEEE J. Biomed. Health Inform..

[20] Chu C.S., Wang D.Y., Liang C.K., Chou M.Y., Hsu Y.H., Wang Y.C., Liao M.C., Chu W.T., Lin Y.T. (2023). Automated Video Analysis of Audio-Visual Approaches to Predict and Detect Mild Cognitive Impairment and Dementia in Older Adults. J. Alzheimer’s Dis..

[21] Jiang Z., Seyedi S., Haque R.U., Pongos A.L., Vickers K.L., Manzanares C.M., Lah J.J., Levey A.I., Clifford G.D. (2022). Automated analysis of facial emotions in subjects with cognitive impairment. PLoS ONE.

[22] Lang P.J., Bradley M.M., Cuthbert B.N. (2008). International Affective Picture System (IAPS): Affective Ratings of Pictures and Instruction Manual.

[23] Bradley M.M., Lang P.J. (2007). The International Affective Digitized Sounds (IADS-2): Affective Ratings of Sounds and Instruction Manual.

[24] Merghani W., Davison A.K., Yap M.H. (2018). A Review on Facial Micro-Expressions Analysis: Datasets, Features and Metrics. arXiv.

[25] Peirce J., Gray J.R., Simpson S., MacAskill M., Höchenberger R., Sogo H., Kastman E., Lindeløv J.K. (2019). PsychoPy2: Experiments in behavior made easy. Behav. Res. Methods.

[26] Jack C.R., Bennett D.A., Blennow K., Carrillo M.C., Dunn B., Haeberlein S.B., Holtzman D.M., Jagust W., Jessen F., Karlawish J. (2018). NIA-AA research framework: Toward a biological definition of Alzheimer’s disease. Alzheimer’s Dement..

[27] Jack C.R., Andrews J.S., Beach T.G., Buracchio T., Dunn B., Graf A., Hansson O., Ho C., Jagust W., McDade E. (2024). Revised criteria for diagnosis and staging of Alzheimer’s disease: Alzheimer’s Association Workgroup. Alzheimer’s Dement..

[28] Frisoni G.B., Festari C., Massa F., Ramusino M.C., Orini S., Aarsland D., Agosta F., Babiloni C., Borroni B., Cappa S.F. (2024). European intersocietal recommendations for the biomarker-based diagnosis of neurocognitive disorders. Lancet Neurol..

[29] Rascovsky K., Hodges J.R., Knopman D., Mendez M.F., Kramer J.H., Neuhaus J., Van Swieten J.C., Seelaar H., Dopper E.G., Onyike C.U. (2011). Sensitivity of revised diagnostic criteria for the behavioural variant of frontotemporal dementia. Brain.

[30] McKeith I.G., Boeve B.F., Dickson D.W., Halliday G., Taylor J.P., Weintraub D., Aarsland D., Galvin J., Attems J., Ballard C.G. (2017). Diagnosis and management of dementia with Lewy bodies: Fourth consensus report of the DLB Consortium. Neurology.

[31] Sachdev P., Kalaria R., O’Brien J., Skoog I., Alladi S., Black S.E., Blacker D., Blazer D.G., Chen C., Chui H. (2014). Diagnostic criteria for vascular cognitive disorders: A VASCOG statement. Alzheimer Dis. Assoc. Disord..

[32] Wilson S.M., Galantucci S., Tartaglia M.C., Gorno-Tempini M.L. (2012). The neural basis of syntactic deficits in primary progressive aphasia. Brain Lang..

[33] Lugaresi C., Tang J., Nash H., McClanahan C., Uboweja E., Hays M., Zhang F., Chang C.L., Yong M.G., Lee J. (2019). MediaPipe: A Framework for Building Perception Pipelines. arXiv.

[34] Russell J.A. (1980). A circumplex model of affect. J. Personal. Soc. Psychol..

[35] Ekman P., Friesen W.V. (1971). Constants across cultures in the face and emotion. J. Personal. Soc. Psychol..

[36] Mollahosseini A., Hasani B., Mahoor M.H. (2017). Affectnet: A database for facial expression, valence, and arousal computing in the wild. IEEE Trans. Affect. Comput..

[37] Cao Q., Shen L., Xie W., Parkhi O.M., Zisserman A. VGGFace2: A Dataset for Recognising Faces across Pose and Age. Proceedings of the 2018 13th IEEE International Conference on Automatic Face & Gesture Recognition (FG 2018).

[38] Ngo Q., Yoon S. (2020). Facial Expression Recognition Based on Weighted-Cluster Loss and Deep Transfer Learning Using a Highly Imbalanced Dataset. Sensors.

[39] keras vggface VGGFace Implementation with Keras Framework. https://github.com/rcmalli/keras-vggface.

[40] Parthasarathy S., Busso C. Jointly Predicting Arousal, Valence and Dominance with Multi-Task Learning. Proceedings of the Interspeech 2017.

[41] Chollet F. (2015). Keras. https://keras.io.

[42] Pedregosa F., Varoquaux G., Gramfort A., Michel V., Thirion B., Grisel O., Blondel M., Prettenhofer P., Weiss R., Dubourg V. (2011). Scikit-learn: Machine Learning in Python. J. Mach. Learn. Res..

[43] Alsuhaibani M., Pourramezan Fard A., Sun J., Far Poor F., Pressman P.S., Mahoor M.H. (2025). A Review of Machine Learning Approaches for Non-Invasive Cognitive Impairment Detection. IEEE Access.

[44] Pellegrini E., Ballerini L., del C. Valdes Hernandez M., Chappell F.M., González-Castro V., Anblagan D., Danso S., Muñoz-Maniega S., Job D., Pernet C. (2018). Machine learning of neuroimaging for assisted diagnosis of cognitive impairment and dementia: A systematic review. Alzheimer’s Dement. Diagn. Assess. Dis. Monit..

[45] Li J., Jin K., Zhou D., Kubota N., Ju Z. (2020). Attention mechanism-based CNN for facial expression recognition. Neurocomputing.

[46] Wen Z., Lin W., Wang T., Xu G. (2023). Distract Your Attention: Multi-Head Cross Attention Network for Facial Expression Recognition. Biomimetics.

[47] Ma F., Sun B., Li S. (2023). Facial Expression Recognition with Visual Transformers and Attentional Selective Fusion. IEEE Trans. Affect. Comput..

[48] Huang Q., Huang C., Wang X., Jiang F. (2021). Facial expression recognition with grid-wise attention and visual transformer. Inf. Sci..

[49] Karthikeyan P., Kirutheesvar S., Sivakumar S. Facial Emotion Recognition for Enhanced Human-Computer Interaction using Deep Learning and Temporal Modeling with BiLSTM. Proceedings of the 2024 5th International Conference on Smart Electronics and Communication (ICOSEC).

[50] Chechkin A., Pleshakova E., Gataullin S. (2025). A Hybrid KAN-BiLSTM Transformer with Multi-Domain Dynamic Attention Model for Cybersecurity. Technologies.

